# Ser ou não ser (da saúde): eis a questão. Reflexões de um cientista social sobre o estar e (quase) pertencer 

**DOI:** 10.1590/0102-311XPT234723

**Published:** 2024-02-26

**Authors:** Élida Azevedo Hennington

**Affiliations:** 1 Escola Nacional de Saúde Pública Sergio Arouca, Fundação Oswaldo Cruz, Rio de Janeiro, Brasil.

“*Odeio as viagens e os exploradores. E aqui estou eu disposto a relatar as minhas expedições. Mas quanto tempo para me decidir! Quinze anos passaram desde a data em que deixei o Brasil pela última vez e, durante todos estes anos, muitas vezes acalentei o projeto de começar este livro; de todas as vezes fui detido por uma espécie de vergonha e de repulsa. Pois será mesmo necessário contar minuciosamente tantos pormenores insípidos, tantos acontecimentos insignificantes?*” ^1^ (p. 11).

Até Lévi-Strauss demorou a superar a “vergonha” ou o “asco” de contar sobre suas expedições, mas, felizmente, o grande pensador acabou nos brindando com um clássico da antropologia. Citar esse trecho da grandiosa obra *Tristes Trópicos*^1^ para abrir essa resenha remonta a ideia de superar o silêncio e o medo, da contribuição de tantas vozes, icônicas ou não, para construir sentidos na vida e de que toda narrativa apresenta uma interpretação autoral, considerando miudezas e insignificâncias, proezas e fracassos, nesse habitar corpo-mundo. Na maioria das vezes, palavras escritas sobre os outros implicados em nós, na tentativa de interpretar o mundo e a nós mesmos.

O livro *Existir e Não Pertencer: Notas Autoetnográficas de um Cientista Social no Campo da Saúde*^2^, de Nelson Filice de Barros, atrai o leitor desde a sua proposta, explicitada nas notas introdutórias: apresentar uma autoetnografia em que o cientista social faz parte do próprio objeto da narrativa. Nesse caso, o livro apresenta uma autorreflexão, que Barros denomina de “prosa”, construída a respeito de uma trajetória de 25 anos como docente e pesquisador, sob o argumento central de “*existir como cientista social no campo da saúde e não pertencer a ele*” (p. 14).

Logo na introdução, o autor remete a autoras e autores, brasileiros e estrangeiros, com os quais dialogou intensamente, como Brah, Anzaldúa, Bauman, Hall, Williams, Hoggart, Thompson, Gilroy, Freire, Luz, Marsiglia e Nunes, que se destacaram como guias regulares no seu percurso em busca de “consensos lastreados nas diferenças”. A narrativa é construída em quatro capítulos: *EU-DIASPÓRICO: Universidade como Espaço de Diáspora*, em que aborda e problematiza a noção contemporânea de diáspora, associada a qualquer grupo que abandona seu lugar de origem; *EU-ESTRANHO: Notas sobre a Construção Social de uma Identificação*, tomando como referência um cientista social “estranho” no campo da saúde, em movimentos de medo e coragem em meio a microinvalidações, ataques e agressões, mecanismos ancestrais de controle e a edificação de seu modo de agir; *EU-AMBIVALENTE: Notas sobre Existir entre Dualidades*, em que discute a falta do “reino social” e o exercício classificatório de produzir leigos e não médicos no campo da saúde, que resultaram na necessidade de eliminar fronteiras para enfrentá-los; *CONHECIMENTO DE FRONTEIRA: Ativos Sociais das Diferenças*, em que discorre sobre como a saúde passou a ser a sua “casa-lar”, ainda que habitando a “terceira margem do rio”. E, por fim, *CODA: A Minha Prosa Não é um “Identikit”*, tendo como últimas palavras uma parte da letra da *Canção do Novo Mundo*.

Autoetnografia ^3^ vem do grego a partir da junção dos termos *auto*, significando próprio, em si mesmo; *ethnos*, como nação, povo, grupo de pertencimento; e *grafo*, forma escrita, grafia. O texto problematiza o fazer, o estar e o pertencer ao campo da saúde de modo a descortinar impressões, reflexões e vivências do corpo-trabalhador, estranho, estranhado, como um ser diaspórico-ambivalente performando numa área que não é a sua. Tomando o seu próprio fazer e a forma de estar no mundo profissional e acadêmico, a escolha do tema e a perspectiva metodológica assumidos por parte do autor despertam o interesse de leitores, especialmente os da área da saúde, ainda pouco familiarizados a produções desse tipo, que colocam a possibilidade de construção de conhecimento baseado numa narrativa autorreferenciada. Desde o início, Barros esclarece o sentido de sua empreitada como “*uma autoescavação que me aproxima de muitos outros corpos que se experimentam em lugares sociais que nunca foram plenamente seus e/ou que eles nunca quiseram pertencer*” (p. 14).

Para Ellis et al. ^4^, a autoetnografia é uma abordagem de pesquisa e escrita que busca analisar a experiência pessoal para compreender a experiência cultural, como um ato político, socialmente justo e consciente. Considerada uma prática anti-hegemônica, está no rol de análises culturais fundamentadas na narrativa pessoal, desenvolvida com “*lente crítica em uma práxis dentro↔fora, de modo a entender quem somos nas nossas comunidades*” ^5^ (p. 71).

Historicamente inserida na tradição interacionista da Escola de Chicago (Estados Unidos) ^3^, a autoetnografia foi atualizada. Segundo Maia & Batista ^6^, o acolhimento do método no Brasil se deu mais recentemente, a partir da entrada, nos centros acadêmicos, de grupos previamente marginalizados devido a seu pertencimento racial, étnico, de identidade de gênero ou de orientação sexual. Incorporados e reconhecidos especialmente devido a políticas afirmativas e de inclusão, mulheres negras e homens negros, quilombolas, indígenas, pessoas LGBTQIA+, entre outras, são hoje uma parcela de pesquisadores encorajados a construir conhecimento de seus grupos sociais interiormente.

“*...A autoetnografia tem a autorreflexão como elemento básico no estudo de grupos sociais em que o pesquisador faz parte de seu próprio objeto e universo de pesquisa. Sua atualidade e interesse resulta de uma transformação política e epistemológica, num contexto pós-colonial ou decolonial, quando indivíduos pertencentes a grupos sociais que antes se constituíam em ‘objetos’ de estudo se transformaram em sujeitos e produtores de conhecimento, gerando um profundo questionamento das bases do discurso científico sobre neutralidade e distanciamento social entre pesquisador e universo de pesquisa*” ^6^ (p. 240).

Barros recorre à autoetnografia performática ^7^ para contar sua trajetória e refletir sobre ela: uma abordagem teórica e metodológica que procura dar voz aos “autores das histórias”, vozes rotineiramente silenciadas e negligenciadas; um método que visa problematizar subjetividades subalternizadas, à margem dos poderes hegemônicos. Em tempos de fortes movimentos decoloniais e identitários, a autoetnografia performática é uma metodologia qualitativa que toma o corpo como lugar privilegiado para a produção de conhecimento:

“*Considerando os pressupostos dos estudos de performance, que compreendem o corpo e suas performances como um local privilegiado para a produção de conhecimento, a autoetnografia performática, um dos tipos possíveis de autoetnografia, apresenta-se como uma estratégia ‘método+lógica’ que promove a quebra de silêncios individuais-coletivos relacionados a sistemas de relações e produção de conhecimento hegemônicos, eurocêntricos, brancos, patriarcais, machistas e cis-heterossexistas*” ^7^ (p. 2).

Como o próprio Barros nos diz, suas anotações, suas lembranças, seus presentes, seus passados, vivos e mortos possibilitaram contar a história de um cientista social no campo da saúde em sua trajetória como ser diaspórico-estranho-ambivalente, num texto etnográfico transformado em ato performático: “*vida, narrativa e melodrama sob os auspícios do capitalismo neoliberal tardio*” ^8^ (p. 2). Norman Denzin é enfático ao defender a autoetnografia performática a partir de uma nova genealogia: “*Não é analítico, nem é profundamente teórico. É mais do que escrita pessoal ou crítica cultural. É mais do que desempenho. Mas é performativo. É transgressor. É resistência. É dialógico. É ético. É político, pessoal, corporificado, colaborativo, imaginativo, artístico, criativo, uma forma de intervenção e um apelo à justiça social*” ^8^ (p. 2).

Barros encontrará o seu público apoiado numa história instigante que não termina; pois, de fato, a cada leitor e a cada leitura, podem surgir novas interpretações. Muitos se reconhecerão (ou não), alguns serão estimulados a compartilhar suas próprias experiências e performar novos conhecimentos, muitos se identificarão e aceitarão o diálogo, embora não sejam cientistas sociais. Afinal, o mundo se move sem fronteiras, apesar da insistência na imposição de barreiras. E ainda que sejamos estranhos um ao outro, um com o outro, somos seres cada vez mais dependentes de compreensão e solidariedade.
